# A Case of Myxoma Arising in the Buccal Mucosa

**DOI:** 10.1155/2021/1358481

**Published:** 2021-08-14

**Authors:** Masahiko Okubo, Yoshie Sano, Yosuke Fukushima, Taketo Tomoda, Shoichiro Kokabu, Tsuyoshi Sato

**Affiliations:** ^1^Department of Oral and Maxillofacial Surgery, Faculty of Medicine, Saitama Medical University, 38 Morohongo, Moroyama-machi, Iruma-gun, Saitama 350-0495, Japan; ^2^Kamimura Dental and Orthodontic Clinic, 3-246-1, Sagami-chou, Koshigaya-shi, Saitama 343-0823, Japan; ^3^Division of Molecular Signaling and Biochemistry, Department of Health Improvement, Kyushu Dental University, 2-6-1 Manazuru, Kokurakita-ku, Kitakyusyu-shi, 803-8580, Japan

## Abstract

Myxomas arising in the oral and maxillofacial areas are extremely rare. This study reports a case of myxoma arising in the soft tissue beneath the buccal mucosa of an 86-year-old man.

## 1. Introduction

Myxomas are benign tumors that usually arise in the heart, skin, bone, or muscle. Cases of the maxillofacial region, in which the main site of occurrence is the maxilla or mandibular bone, are relatively rare [1]. Here, we report a case of myxoma arising in the soft tissue beneath the buccal mucosa.

## 2. Case Report

**Figure 1:**
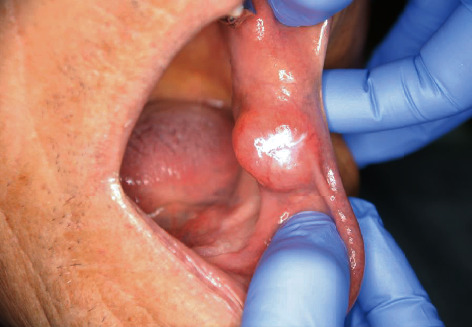
Intraoral clinical findings. The mass was round and located in the left buccal mucosa. The surface showed normal mucosa.

**Figure 2:**
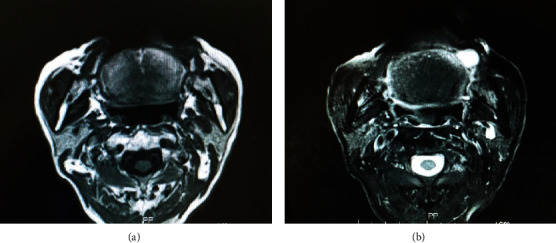
Magnetic resonance imaging of the tumor. (a) T1-weighted MRI showed a mass with low intensity in comparison with skeletal muscle tissue (yellow dot) while (b) T2-weighted MRI revealed a lesion with high intensity in comparison with adipose tissue (yellow dot).

**Figure 3:**
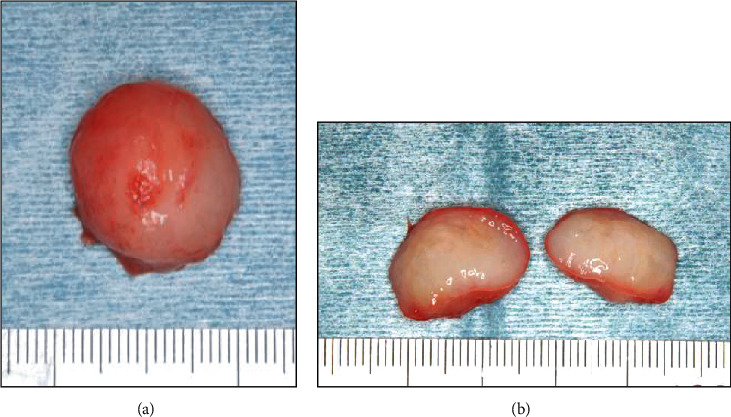
Gross appearance of the resected specimen (a). The surgical specimen measured 2 cm at its largest diameter and was filled with glossy, mucoid, and milky white secretions (b).

**Figure 4:**
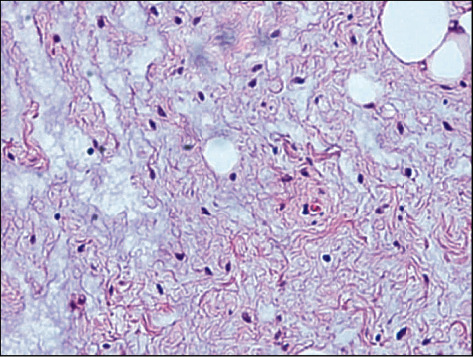
Histopathological findings. On hematoxylin and eosin staining, tumor cells were present in the sparse mucoid matrix. The original magnifications were ×200.

An 86-year-old male was referred to Saitama Medical University Hospital (Moroyama, Saitama, Japan) from a local dentist in July 2015 due to the presence of an intraoral mass. The patient had not noticed the mass until the dentist brought it to his attention. His previous history included transitional cell cancer of the bladder, but his family history was unremarkable. The round mass, measuring 12 mm in diameter, was located in the left buccal mucosa (Figure 1). The mass was soft, nontender, and nonadherent to the surrounding tissue, and the surface exhibited normal mucosa. Magnetic resonance imaging (MRI) of the left buccal region showed a mass with intensity lower than that of skeletal muscle tissue on T1-weighted imaging (Figure 2(a)) and with intensity higher than that of adipose tissue on T2-weighted imaging (Figure 2(b)). This was diagnosed as a benign buccal tumor and was subsequently resected under local anesthesia. The tumor was easily dissected from the surrounding tissue and appeared to be covered with a thin fibrotic capsule. The resected specimen measured 2 cm at its largest diameter (Figure 3(a)) and was filled with glossy, mucoid, and milky white materials (Figure 3(b)). Microscopically, the tumor cells in the sparse mucoid matrix (Figure 4) exhibited a spindle or stellate shape without nuclear atypia (data not shown). The tumor matrix had abundant collagen fibers and was strongly stained with alcian blue, indicating the presence of acidic polysaccharides (data not shown). On immunohistochemistry, the tumor cells were negative for S-100 protein, ASMA, and CD3 (data not shown). The final histopathological diagnosis was a myxoma. The tumor was completely resected, and five years later, the patient exhibited no recurrence or distant metastasis.

## 3. Discussion

Myxoma is a relatively rare benign tumor derived from soft tissue and was first described by Virchow as a tumor histologically resembling the mucinous substance of the umbilical cord. Myxoma was defined as a neoplasm without mesenchymal cells, such as chondrocytes, myoblasts, or adipocytes, and without metastatic capacity [1].

Myxomas most frequently occur in the myocardium, followed by the subcutaneous and intramuscular regions of the lower leg or shoulder. Myxomas arising in the oral and maxillofacial areas are extremely rare. Moreover, most of these cases occur in the maxillary or mandibular bones as odontogenic myxoma derived from odontogenic cells. Thus, only a few reports on soft tissue myxomas are available in the literature [1].

Patients usually recognize a slowly growing indolent mass for myxomas occurring in the soft tissue of the oral and maxillofacial regions. Following gradual enlargement, functional disorders such as difficulty in chewing and/or swallowing may develop, depending on the location [2]. In our case, the patient was asymptomatic and did not recognize the mass until it was noticed by his dentist.

Myxoma usually appears as a mass with smooth margins, with low intensity on T1-weighted MRI and with high intensity on T2-weighted MRI. However, these are not features specific to myxomas and are relatively common in benign soft tissue tumors. The same findings were present in our patient, leading us to a clinical diagnosis of a benign tumor in the soft tissue.

In this case, cytoscreening was not performed. Generally, cytology by fine needle aspiration cannot be used to diagnose myxomas since the tumor has few cellular components. Histopathological analysis revealed spindle-shaped tumor cells with pyknotic nuclei and elongated protrusions, sparsely arranged in a matrix containing mucoid substances. Immunohistochemistry showed that the myxoma cells were positive for vimentin, an intermediate filament specific to mesenchymal cells, and were negative for desmin and S-100 protein [3]. In addition, immunohistochemistry of other proteins may have some utility in investigating differential diagnoses of adipoma, chondroma, fibroneuroma, or myxoid liposarcoma [4]. The treatment of myxoma is essentially surgical resection. Many cases of myxoma do not have thick capsules, resulting in a tendency to expand and invade the surrounding tissue. Therefore, myxomas should be resected with normal surrounding tissue to prevent recurrence [5].

## 4. Conclusion

We encountered a case of myxoma arising in the buccal mucosa. Myxomas have no metastatic capacity but may occasionally recur. Thus, careful long-term follow-up is needed.

## Data Availability

All the data are presented in the paper.
